# Status of animal health biosecurity measures of dairy farms in urban and peri-urban areas of central Ethiopia

**DOI:** 10.3389/fvets.2023.1086702

**Published:** 2023-03-29

**Authors:** Nebyou Moje, Hika Waktole, Rediet Kassahun, Bekele Megersa, Milkessa T. Chomen, Samson Leta, Mulu Debela, Kebede Amenu

**Affiliations:** ^1^College of Veterinary Medicine and Agriculture, Addis Ababa University, Bishoftu, Ethiopia; ^2^Department of Agricultural Economics, Ambo University, Ambo, Ethiopia; ^3^Department of Rural Development and Agricultural Extension, Ambo University, Ambo, Ethiopia; ^4^Animal and Human Health Program, International Livestock Research Institute, Addis Ababa, Ethiopia

**Keywords:** animal health, biosecurity, management practices, smallholder dairy farm, urban and peri-urban

## Abstract

Ethiopian dairy farming has many constraints including disease and lack of appropriate biosecurity measures. With this into consideration, a cross-sectional survey was carried out from November 2021 to April 2022 to determine the animal health biosecurity status of dairy farms and investigate the sociodemographic characteristics of livestock keepers on dairy farm management. A face-to-face questionnaire survey using an online application was used to collect data. The interview involved a total of 380 dairy farms located in six towns in central Ethiopia. The results showed that out of the surveyed farms, 97.6% missed footbaths at their gate points, 87.4% lacked isolation areas for either sick or newly introduced cattle, and 83.4% did not check the health status or quarantine newly introduced cattle. Furthermore, written formal record-keepings on animal health was uncommon, except for a few farms (7.9%). However, nearly all of the respondents (97.9%) gave medical treatments for sick cattle, and 57.1% of them vaccinated their herds regularly during the past 12 months before the survey. Hygienic aspects of the farms showed that 77.4% of the dairy farms appeared to clean the barn on a daily basis. However, 53.2% of respondents did not utilize personal protective equipment while cleaning their farms. A quarter of the dairy farmer (25.8%) avoided mixing their cattle with other herds, and 32.9% of them have implemented isolation of sick animals. In general, the animal health biosecurity assessment of the farms showed that most of the dairy farms (79.5%) earned unacceptable biosecurity levels (score of ≤ 50%), whereas the remaining 20.5% of dairy farms had received a score of >50% (“acceptable level”). The gender of dairy farmers (χ^2^ value = 7.61; *p* = 0.006), education level (χ^2^ value = 12.04; *p* = 0.007), dairy farm ownership (χ^2^ value = 41.6; *p* < 0.001), training on dairy farm management (χ^2^ value = 37.1; *p* < 0.001), towns (χ^2^ value = 31.69; *p* < 0.001), farm size (χ^2^ value = 7.7; *p* = 0.006), and herd size (χ^2^ value = 28.2; *p* < 0.001) showed a significant statistical association with biosecurity status. Finally, the study revealed that the level of biosecurity adoption of dairy farms in central Ethiopia is mostly unsatisfactory and calls for designing and implementing intervention measures toward improved animal health in dairy farms and further public health.

## Introduction

Disease prevention at a herd level has become increasingly important in modern veterinary practice compared with individual animal healthcare. The implementation of biosecurity is part of the paradigm shift from treating individual animals to preventing diseases from entering the animal population (1). Biosecurity is defined as a set of management techniques that prevent disease agents from entering the farm (external biosecurity) while also limiting disease agents' spread within the herd (internal biosecurity) (2, 3). In addition, it encompasses animal health management, isolation, and premise sanitation, all of which are frequently used to assess a farm's overall biosecurity practices (4).

Adopting acceptable biosecurity practices is the most cost-efficient and effective disease prevention and control method available in the modern herd management approach (5). In this regard, identifying the risks presented by key infectious pathogens and minimizing those risks through diverse management strategies are essential components of a biosecurity program. This demands knowledge of biosecurity principles and the purpose of disease prevention (6). Despite these biosecurity advantages, dairy farmers rarely implement biosecurity control measures on their farms (7). In some cases, insufficient attention to biosecurity implementation may have a significant adverse effect on animal health and welfare (8), resulting in a financial loss (9) and a public health concern (10).

Traditional extensive and modern (intensive) dairy systems are the two main types of cow-based dairy production systems in Ethiopia (11). Modern dairy production involves keeping relatively large herd sizes of exotic or crossbred cattle under intensive or semi-intensive management with more inputs such as the use of agro-industrial byproducts-based feeds, implementing proper animal health management, and the application of biosecurity measures. On the other hand, traditional smallholder dairy farms are pasture-based; mostly indigenous cattle are kept for subsistence production with minimal supplementation of feeds and the absence of biosecurity measures. In Ethiopia, combined milk production from the two systems in 2020/2021 was estimated to be 4.03 billion liters with a higher contribution from the traditional system (88%) than the modern dairy farms (12%) (12). Moreover, dairy farming is rapidly expanding, involving a large number of small- and large-scale, market-oriented farms (13). The major difference between large and smallholder farms is their herd size. Furthermore, the management aspects usually differ, as the smallholder follows a semi-extensive system and keeps local breeds with low input. Other than this difference, a large-scale farming system is resource intensive and demands a lot of land, labor, housing, health management, and other infrastructure (e.g., water supply) (14). The performance of the dairy herd, however, is hindered by different factors, including feed scarcity (both quantity and quality of feed), the low genetic potential of local breeds, incidence of diseases and parasites, ineffective management practices, a lack of competent health services, and technological support (15).

Research regarding biosecurity measures was previously conducted in the central part of Ethiopia, though limited to the feedlot farming system (16). Taking the vital importance of biosecurity levels to the farm enables the implementation of emerging disease contingency planning and strengthens disease prevention strategies (17). Nonetheless, information is scarce in the study area regarding the status of biosecurity measures in the dairy sector. Hence, the current study was designed to assess the current status of biosecurity measures on dairy farms in the area. In addition, understanding the relationship between the demographic characteristics of dairy farm owners (determinants) and the biosecurity status of the dairy farms in central Ethiopia is vitally important in highlighting any potential intervention areas.

## Materials and methods

### Study area

**Figure 1:**
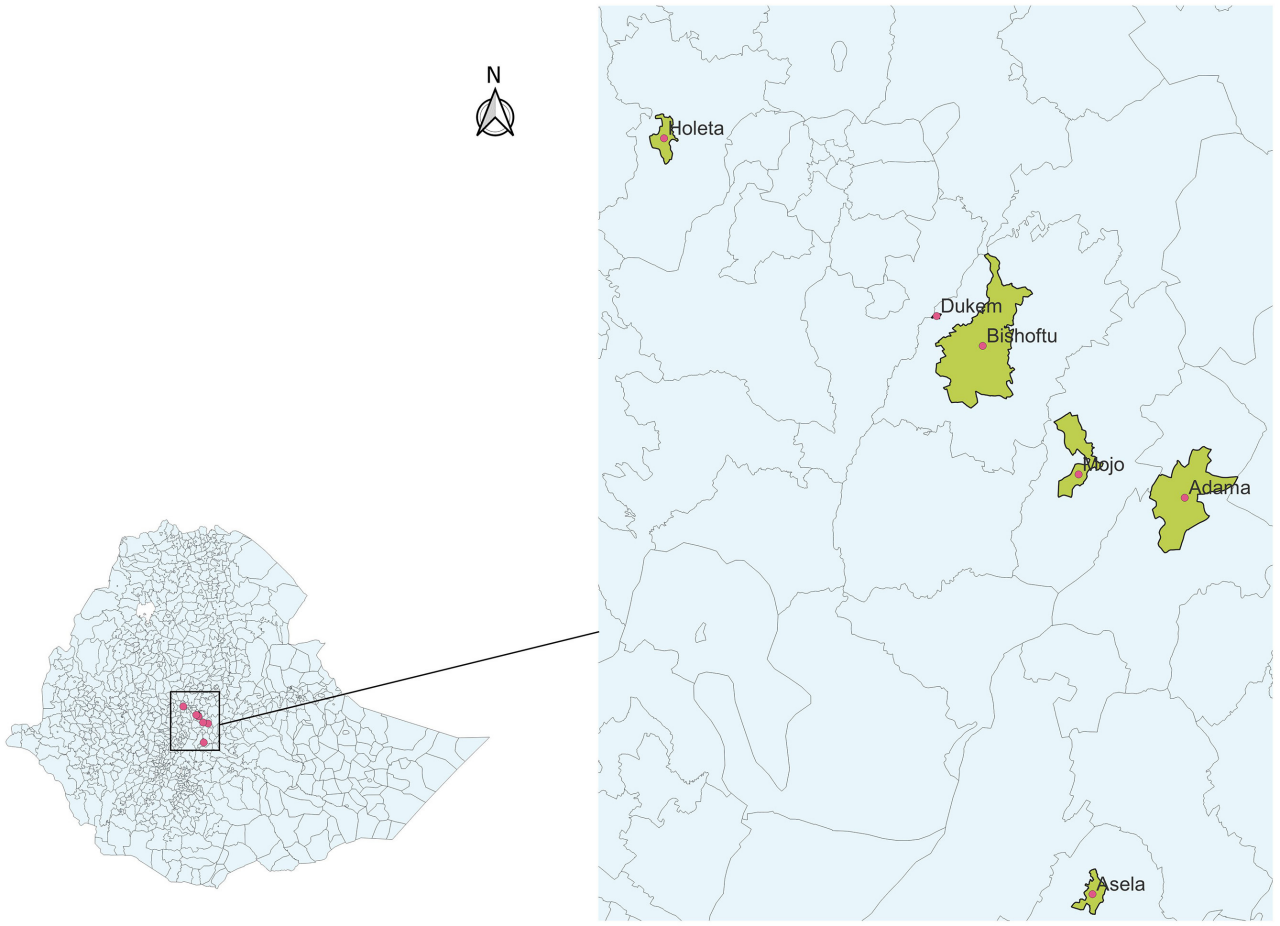
Map of Ethiopia showing the relative location of the towns of the study farms.

The current study was carried out on different dairy farms from Adama, Asela, Mojo, Bishoftu, Dukem, and Holeta towns located in central Ethiopia (Figure 1). Dairy cattle for some specific places, such as Adama, Mojo, Bishoftu, and Dukem, were expressed in a single zone of East Shewa, where they are the major places known for dairy farming. This was made due to a lack of information for the specific places, while for the rest of the study sites, town-specific cattle population was given (Table 1).

**Table 1:** Basic information on cattle, human populations, and agro-ecology of study areas.

**Study areas**	**Distance from Addis Ababa**	**Location (coordinates)**	**Elevation (m)**	**Average Annual temperature (°C)**	**Average Annual rainfall (mm)**	**Cattle populations**	**Human populations**
Adama	100 km East	8°33′N 39°27′E	1,700 m	20.7	897.9	78,957 dairy cows (this figure stands for the East Shewa zone where the abovementioned towns are mainly known for having significant dairy cows)	435,222
Bishoftu	47 km Southeast	8°45′N 38°59′E	1,850 m	12.3–27.7	800		197,557
Dukem	37 km South	8°45′N 38°56′E	2,100 m	23 and 26	963		13,184
Mojo	66 km Southeast	8°39′N 39°5′E	1,790 m	19	967.9		58,406
Holeta	29 km West	9°3′N 38°30′E	2,400 m	15.7	1,037.9	185,000 (only 2% are cross-bred dairy cows = 3,700)	46,041
Asela	175 km South	8°49′N 40°41′E	2,500–3,000 m	8.4–22.6	2,000	324,000 (14,000 cross-bred dairy cows)	132,926

Source: [18–20] and other sources (https://www.statsethiopia.gov.et/wp-content/uploads/2020/08/Population-of-Towns-as-of-July-2021.pdf).

### Study design

A cross-sectional survey was carried out from November 2021 to April 2022. Individual small-scale producers, small-scale cooperatives, and commercial farms make up the target farms in the study towns.

For the sample size calculation, it was assumed that 50% of the dairy farms may meet the required biosecurity measures (18), and the calculation was carried out based on the following formula:


$$\begin{array}{l} n=\frac{Z^{2}\;\times \;P\;\left(1-P\right)}{d^{2}}, \end{array}$$

where *Z* = 1.96 (95% confidence level), *d* = marginal error to be 0.05, *P* = proportion of interest assumed to be 50%, and 1 – *P* = proportion of farmers not meeting the required biosecurity level.

Study sites (towns) were selected purposefully based on different criteria, such as the abundance of dairy farms [as mentioned by Melese and Jemal (19)], access to the premises *via* transportation means, and the security situation for the area. The towns considered in the present study were Asela, Adama, Mojo, Bishoftu, Dukem, and Holeta. We believe that the towns can be representative of the urban and peri-urban dairy production systems of central Ethiopia. Furthermore, the specific dairy farms in those selected sites were selected with the help of local animal health professionals and experts working on other livestock extensions, such as biogas technology, to get representative samples.

The absence of complete lists of dairy farmers in each town hindered the random selection of the farmers. Therefore, a kind of “snowball sampling” was employed instead of full randomization. The farm owners were requested to participate in this study after explaining its main objectives, which were finally confirmed with their verbal consent. If owners did not consent to participate in this study, the next dairy farms would be taken after following the same consent protocols. Only farmers who consented to participate in this study were included. The respondents were asked for their willingness to participate while maintaining confidentiality and could withdraw from the study at any time.

### Data collection tools and methods

Data were collected through face-to-face interviews using a structured questionnaire with open-and closed-ended questions. The current questionnaire format was adopted from previous studies (1, 2, 6, 10, 16) and further refined by considering the local context. Finally, the questionnaire was imported into an online data collection application (KoboToolbox, https://www.kobotoolbox.org). Ethical clearance was gained from the College of Veterinary Medicine and Agriculture of Addis Ababa University (CVMA-AAU) for the content of the questionnaire, and appropriate corrections from the ethical committee were incorporated. The questionnaire was designed in English and subsequently translated by the interviewer into the local language (Amharic or Afan Oromo depending on the preference of the respondent) when interviewing respondents. The draft version of the questionnaire was pretested with three dairy farm owners from each town who were not included in the final sample. Pre-testing was utilized to revise and rephrase unclear questions. Contents of the questionnaire elicit information about respondents' sociodemographic factors, isolation, sanitation, and animal health management which was prepared to gather data on biosecurity practices. Respondents in this study were all dairy farm owners, and in this article, respondents and owners are used synonymously.

The determination of the adoption level of biosecurity measures was made with a quantitative scoring system. This system was designed based on the assumption that all prospective biosecurity practices weighed equally, ranging from 0 to 1. A biosecurity practice was coded as 1 if the practice was applied or 0 if not implemented (20, 21). A total of 10 biosecurity practices at dairy farms [i.e., use of foot baths, presence of isolation pens, quarantining practice, dairy cow treatment, vaccination, record keeping, routine pen cleaning, use of personal protective equipment while cleaning, avoidance of mixed herds, and isolation of sick animals (i.e., from public health perspectives)] were adopted from the study of Can and Altug (21) and included in this study to evaluate a component of biosecurity status. Each farm biosecurity practice received a score that varied from 1 to 10 (corresponding to several correct practices), with a higher number indicating a higher level of biosecurity measure. Finally, the adoption level of biosecurity practices by farmers was obtained by dividing the number of practices the farmers applied by the total number of practices. In total, 10 major animal health biosecurity practices for animal health were considered. This proportion was expressed as a percentage, which was later categorized as “unacceptable” and “acceptable” biosecurity level when the score appeared to be <50 and >50%, respectively.

### Data analysis

The data were entered into a Microsoft Excel spreadsheet, followed by data cleaning and coding. Descriptive statistics were obtained to calculate frequencies and percentages of different factors. Further statistical analysis was conducted using STATA version 15 to look at the association between the demography of farm owners and farm characteristics with biosecurity status by performing Pearson's chi-square test (χ^2^). Statistical significance was judged to exist with a *p*-value of ≤ 0.05.

## Results

### Dairy farm characteristics

Out of 384 farms targeted, data collected from four farms had some data quality issues and were discarded in the end, and results were based on 380 farms. In this study, land was found to be an important resource for various aspects of dairy farming. In this regard, the survey findings showed ~135 (35.5%) farms established in an area < 60 m^2^, 151 (39.7%) had a size of ≥60 m^2^, and the remaining 94 (24.7%) did not disclose such information. In terms of herd size, the majority of dairy farms (60.3%) had less than four cows while the rest (39.7%) owned four and more cattle. Dairy cows kept on the farm range from cross-bred or exotic to local breeds (Table 2), with most of the farms keeping cross-bred cattle (75.3%), followed by those who keep both local and cross-bred (15.5%) and local breeds only (9.2%).

**Table 2:** Characteristics of the study dairy farms in central Ethiopia (*n* = 380).

**Characteristics**	**Categories**	**Number (%)**
Farm location	Adama	70 (18.4)
	Asela	114 (30)
	Bishoftu	81 (21.3)
	Dukem	20 (5.3)
	Holeta	63 (16.6)
	Mojo	32 (8.4)
Farm size (m^2^)	< 60	135 (35.5)
	≥60	151 (39.7)
	Not determined	94 (24.7)
Herd size	≤ 4 (small)	229 (60.3)
	>4 (large)	151 (39.7)
Breed of cattle	Local	35 (9.2)
	Cross-bred	286 (75.3)
	Both breed	59 (15.5)

### Demographic characteristics of dairy farm owners

Out of the 380 dairy farmers surveyed, 57.4% were women and 79.8% of them were aged older than 51 years. The educational backgrounds of the respondents varied from those without formal education to college studies. Approximately one-third (31.6%) have completed primary school, and others did secondary (29.5%) and college (14.7%) studies, while a quarter of the respondents (24.2%) had no formal education. Nearly all farms, except for cooperatives (4.2%) and private companies (2.4%), were owned by family members (either household heads or members). Thus, dairy farming was found to be the main source of livelihood for most dairy farmers (60.3%) and their families. Only a quarter of the farmers had training in dairy farm management, while a majority (72.4%) of them neither had such training nor received any formal information about the operation of dairy farms (Table 3).

**Table 3:** The demographic characteristics of dairy respondents in central Ethiopia (*n* = 380).

**Variables**	**Category**	**Number (%)**
Gender of respondent	Male	162 (42.6)
	Female	218 (57.4)
Age of respondents	≤ 51	303 (79.7)
	>51	77 (20.3)
Education level	No education	92 (24.2)
	Primary education	120 (31.6)
	Secondary education	112 (29.5)
	College	56 (14.7)
Dairy farm ownership	Family	356 (93.7)
	Cooperatives	16 (4.2)
	Private company	8 (2.1)
Dairy as a primary source of income	No	151 (39.7)
	Yes	229 (60.3)
Training on dairy farm management	No	275 (72.4)
	Yes	105 (27.6)

### Dairy farm biosecurity measures

Among the dairy farms that were assessed, only 2.4% had a footbath at their farm entrance point, and 12.6% owned quarantine facilities for newly arriving animals before being introduced animals to the existing herd. Further inquiry regarding the introduction of new animals showed that 83.4% of dairy farms never checked the health status of the incoming cattle into farms. They mostly adopted healthcare such as treatment of sick animals for general health concerns was regularly (98%) practiced, and 57.1% of the dairy farms vaccinated their cattle within the last 12 months before the survey. The status of record keeping by dairy farms (i.e. cattle information and health records) were observed in few (7.9%) farms and the rest lacks this important information in their record sheet.

A sanitation status assessment of dairy farms showed that 77.4% of the respondents reported a daily cleaning of the cattle pen though more than half (53.2%) of them did not take any precautions (personal protective clothes, i.e., hand gloves, face, and nose mask, and boots) during cleaning. On the other hand, only 25.8% of surveyed farms avoid mixing herds with other animals, while 32.9% of the respondents indicated that sick and suspicious animals were handled separately (Table 4).

**Table 4:** Biosecurity measures taken at different dairy farms in central Ethiopia (*n* = 380).

**Biosecurity measures**	**Yes (%)**	**No (%)**
Use of footbath at the entrance of farm	9 (2.4)	371 (97.6)
Presence of dedicated isolation place for sick animals	48 (12.6)	332 (87.4)
Checking health status of newly arrived animals to the farm	63 (16.6)	317 (83.4)
General treatment of sick animals	372 (97.9)	8 (2.1)
Vaccination with last 12 months	217 (57.1)	163 (42.9)
Record keeping (on medical treatment, vaccination)	30 (7.9)	350 (92.1)
Consistent daily cleaning of cattle pen	294 (77.4)	86 (22.6)
Precaution taken while cleaning the pen (including use of personal protective equipment such as gloves and rubber boots)	178 (46.8)	202 (53.2)
Avoid mixing of animals from other herd and livestock species	282 (74.2)	98 (25.8)
Separation of sick animals consistently applied on the farm	125 (32.9)	255 (67.1)

### Factors associated with biosecurity practices

The present study showed a low level of overall biosecurity status of dairy farms with most of the farms, [302 (79.7%)] farms earned a score of ≤ 50%, which was regarded as “unacceptable” biosecurity practices. The remaining 78 (20.3%) of the farms had a score of >50%, hence considered as “acceptable” (Table 5), which was used to create a binary dependent variable of the dairy farm biosecurity status.

**Table 5:** Biosecurity security score level (%) of the dairy farms in central Ethiopia.

**Number of farms**	**Percent of farms**	**Biosecurity score (out of 10)**	**Biosecurity score level (%)**	**Category**
7	1.8	1	10	Unacceptable (79.7%)
38	10.3	2	20	
92	24.2	3	30	
94	24.7	4	40	
71	18.7	5	50	
42	11.1	6	60	Acceptable (20.3%)
20	5.3	7	70	
6	1.6	8	80	
4	1.1	9	90	
5	1.3	10	100	

The sociodemographic factors of respondents and farm characteristics were assessed for the presence of association with farm biosecurity status using Pearson's chi-square test. Of these characteristics, the gender of the respondents (χ^2^ value = 7.61; *p* = 0.006), education level (χ^2^ value = 12; *p* = 0.007), dairy farm ownership (χ^2^ value = 41.6; *p* < 0.001), training status on dairy farm management (χ^2^ value = 37.1; *p* < 0.001), dairy farm location/site (χ^2^ value = 31.7; *p* < 0.001), farm size (χ^2^ value = 7.7; *p* = 0.006), and herd size (χ^2^ value = 28.2; *p* < 0.001) showed statistically significant association with biosecurity status. Improved and acceptable biosecurity measures were observed with those dairy farms owned by collage education and who took training on dairy farm management. Dairy farms located in Bishoftu were observed to have acceptable biosecurity, while Mojo and Asela had the lowest percentage category for acceptable biosecurity measures. The other factors with a relatively higher percentage of acceptable biosecurity measures were dairy farms from cooperatives (owned by cooperatives organized with a group of individuals owning dairy farms), with large space for routine activities of the dairy operation (e.g., milking, feeding, etc.), and large herd sizes than others (Table 6).

**Table 6:** Association between sociodemographic variables and dairy farm biosecurity status.

**Variables**	**Categories**	**No**.	**Biosecurity compliance (%)**		**Pearson's chi square test**	
			**Acceptable (%)** ***n*** = **78**	**Poor (%)** ***n*** = **302**	χ^2^ **value**	***P*** **-value**
Gender of respondent	Male	162	44 (27.2)	118 (72.8)	7.61	0.006
	Female	218	34 (15.6)	184 (84.4)		
Age of respondent	≤ 51	303	65 (21.5)	238 (78.5)	0.78	0.375
	>51	77	13 (16.9)	64 (83.1)		
Education level respondent	Illiterate	92	15 (16.3)	77 (83.7)	12.04	0.007
	Primary	120	20 (16.7)	100 (83.3)		
	Secondary	112	22 (19.6)	90 (80.4)		
	College	56	21 (37.5)	35 (62.5)		
Dairy farm ownership	Family	356	62 (17.4)	294 (82.6)	41.60	< 0.001
	Cooperatives	16	8 (50)	8 (50)		
	PLC	8	8	0		
Dairy as primary income	No	151	33 (21.8)	118 (78.2)	0.27	0.603
	Yes	229	45 (19.6)	184 (80.4)		
Training on dairy farm management	No	275	35 (12.7)	240 (87.3)	37.10	< 0.001
	Yes	105	43 (41)	62 (59)		
Town	Adama	70	9 (12.9)	61 (87.1)	31.69	< 0.001
	Asela	114	12 (10.5)	102 (89.5)		
	Bishoftu	81	32 (39.5)	49 (60.5)		
	Dukem	20	5 (25)	15 (75)		
	Holeta	63	17 (27)	46 (73)		
	Mojo	32	3 (9.4)	29 (90.6)		
Farm size (m^2^)	< 60 (small)	135	23 (17)	112 (83)	7.65	0.006
	≥60 (large)	151	47 (31.1)	104 (68.9)		
	Not determined	94	8 (8.5)	86 (91.5)		
Cattle herd size	< 4 (small)	165	13 (7.9)	152 (92.1)	28.16	< 0.001
	≥4 (large)	213	64 (30)	149 (70)		
Cattle breed	Local	35	4 (11.4)	31 (88.6)	2.29	0.317
	Crossbred	286	63 (22)	223 (78)		
	Both	59	11 (18.6)	48 (81.4)		

PLC, private limited company; ND, not determined.

## Discussion

The present study identified that a small proportion (2.4%) of the dairy farm used footbath at their farm gate. This is in agreement with a recent report (22) in which most farmers identified the approach as beneficial but not applied in dairy farms, unlike its compulsory application to poultry farms. Dairy barn cleaning practices showed that a higher proportion of dairy farmers (77.4%) cleaned their barns daily, which contributes to disease prevention. A similar study finding by Tegegne and Tesfaye (23) also showed that 88.3% of dairy farms cleaned the barn on daily basis. On the contrary, Abayneh et al. (24) reported a lower percentage (31.4%) of the farms compared to current findings which implemented daily cleanings and attributed their finding to the lack of water. More than half (53.2%) of respondents did not use protective equipment while cleaning the pen similar to the observation of Jaswal et al. (25), in which pen cleaning was performed by bare hands. Such practice is a critical pathway for pathogen transfer from animals to humans, specifically when women, who do most of the cleaning activities with bare hands, are engaged in food preparation and feeding children without proper hand washing using water and detergents. Cattle are also major reservoirs of pathogenic organisms such as *Escherichia coli*, coliforms, *Leptospira, Salmonella*, and *Cryptosporidium*, for which young children are at a greater risk of infection, leading to increased incidence of diarrhea and malnutrition (26).

In the current study, ~16.6% of respondents used quarantine facilities where the health status of cattle could be checked and animals were monitored before they were introduced to the rest of the animals in the herd. Another study from Belgium also reported ~12% of the farms used quarantine for their newly introduced cattle (27). A much lower figure has been also reported (28) from the central highlands of Ethiopia in which only 4% of the farms used a quarantine scheme for newly introduced cattle. This could be related to either due to lack of awareness of the importance of quarantine or a lack of separate facilities for quarantine purposes on their farms, as most farms have limited space. Though the quarantine facility was reported lower (16.6%), the majority of the farms (67.1%) in this study practiced isolation of sick animals which was negligible as already reported by Edwards who made his study in feedlot cattle (29). However, Gizaw et al. (30) stated the existence of a higher percentage of farms (74.2%) reported mixing their herds with other herds at grazing areas and watering points. The authors also stated that their study farms keep their cattle mostly on an extensive management system which is not the case in the present study in which indoor feeding is common.

Even though testing or diagnosing animals for infectious disease upon arrival at the farm is a basic disease prevention approach, the majority (83.4%) of farms in this study did not check the health status of animals they brought to their herd. The reason for not testing newly introduced animals may be due to a lack of kits or expertise, and it requires contacting either private or public veterinary services. Thus, farm owners do not opt for testing the animals for any disease upon arrival, rather they prefer buying dairy cattle from a known source for which information about the animal is available.

The survey showed that treatment of diseased animals was performed by a majority of dairy farms (97.9%), and more than half of the farms (57.1%) have vaccinated their cattle in the last 12 months before the study period. However, Duguma (31) reported the practice of vaccination following the disease outbreak incidents in the respective areas of the study. Some aspects of the information could be missed as only a few farms keep health records (7.9%), similar to the finding of Duguma (31) who reported 5% for the farms in southwestern Ethiopia. On the other hand, Can and Altug (21) reported a higher percentage (36%) of animal health record-keeping practices by small-scale farmers in Hatay, Turkey. This may indicate the need for awareness creation among farmers on the importance of record keeping regarding health status and intervention measures.

In contrast to our observation, Assan (32) reported that dairy farming is more likely practiced by male than female farmers. This was backed by the idea of socioeconomic and cultural biases which have reduced the role of women in livestock production (15, 33). In the current study, however, female-owned dairy farms were more than half (57.4%) of the sample which is in disagreement with that of Firdissa et al. (34) (21.5%). In the current study, unacceptable biosecurity compliance (84.4%) was observed in female dairy farm owners which could be related to women's lower literacy rate (16%) compared to that of men in Ethiopia (35). Low literacy could hinder women's ability to conduct required biosecurity practices. Furthermore, educational level assessment with regard to biosecurity compliance showed higher acceptable biosecurity compliance with farmers with college degrees (37.5%), as compared to others. This is actually explained by the fact that education enhances people's ability to search for and assimilate information, which leads to a greater willingness to accept and adopt new procedures or technology for the benefit of their business. In this regard, much work has to be carried out to encourage herd owners to enhance their educational position in order to make informed farm decisions (36).

The dairy farm owned by the cooperative had a significant level of higher biosecurity implementation (*p* < 0.001) than others. This variation could be correlated with the country's socioeconomic condition, particularly for smallholder farmers with low bargaining power, skills, and expertise (37). Thus, dairy cooperatives are often regarded as a critical foundation, enabling farmers to resolve the barriers that prevent them from taking advantage of the opportunities associated with acceptable dairy management (38, 39). As a result, cooperatives provide lower treatment costs, increased accessibility of vaccination, and thus enhance farmers' adoption of biosecurity measures through training (40–42).

Assessment of training on dairy management showed that a majority (72.4%) of the farmers had not obtained any training regarding dairy management which gave rise to a significant variation with biosecurity implementation (15). Thus, this could be accountable for the lower adoption of biosecurity practices (28, 43). The adoption of enhanced biosecurity practice was positively associated with dairy farmer training and knowledge of dairy husbandry practices (44). Hence, it is essential to promote training opportunities concerning dairy production and basic husbandry practices.

There was a spatial association between dairy farm locations and the level of biosecurity measures. This could be due to the differences in the accessibility to the source of knowledge, training, and technical assistance at these locations (source: informal communications), which agreed with the finding of Sayers et al. (45). Towns, such as Bishoftu and Dukem, are closer to various institutions, such as CVMA-AAU, Agriculture Research Center, and National Veterinary Institute, which were found in Bishoftu, which have better biosecurity status than others. Professionals working in those institutes might directly or indirectly contribute to the improved farm biosecurity measures through one wing of their mission, community engagement/service.

Of the respondents, the majority of the farmers had fewer cows and were found to negatively affect the level of biosecurity practice which could be subjected to generalization due to the absence of sufficient large-sized farms. This can be actually related to the fewer large-sized farms and more importantly related to the reduction of the number of dairy cows due to the existing animal feed shortage and higher costs. Otherwise, large-sized farms are perceived to better address cattle health issues through investing in the best herd health practices due to the huge investment in the sector which was not the case in most of the smallholder dairy farms. This reflects greater adoption of biosecurity practices on larger dairy farms compared to smaller farms (46). This can be related to the higher economic investment on large farms with a potential capital to invest on farm healthcare and management. It is, however, important to note that the larger the herd size, the higher the population density and herd dynamics, contributing to the increased occurrence of diseases. Similarly, Eguale et al. (47) noted that the increased herd size puts the farmer at risk of introducing diseases, as well as spreading within the farm, as it enhances animal-to-animal contact.

The study showed that 39.7% of the studied dairy farms were established on land size ≥60 m^2^. However, the researcher's observation notes a higher stocking density in the study sites that leads to overcrowding in some dairy farms. Hordofa et al. (48) explained that overcrowding of dairy cows has a negative effect on the health status of cows. In addition, Duguma (31) stated that owners could face problems with dairy farm expansion due to insufficient space for dairy operations (including where animals are kept and various activities such as milking and feeding of animals carried out). Thus, biosecurity scores were significantly associated with the farm size.

### Limitations of the study

The present study is not without limitations. A lack of standardized biosecurity criteria customized for smallholder dairy farms made it difficult to cover detailed biosecurity information about dairy farms holding few animals. The other limitation is related to the absence of updated complete lists of dairy farms in each town to allow for selection using a randomization process that can also impact confounders. Instead, a “snowball” type sampling strategy was followed which is a non-probabilistic type. As a result, only descriptive statistics were calculated by limiting the use of other statistical tools. However, it is believed that this study can still provide useful baseline information about dairy health biosecurity measures for potential interventions toward improved practices. The other aspects related to biosecurity scoring as a dichotomous category could lead to some level of biasness related to a close percentage but are believed to at least show the general category of biosecurity level. Since 2015 (49), the dairy sector, a priority livestock sub-sector of the Ethiopian government, and the present study can be a useful input toward dairy health improvement initiatives.

## Conclusion

This study has provided a better understanding of the relationships between dairy farm owners' sociodemographic variables, farm features, and biosecurity status of the dairy farms in six towns in Ethiopia. The gender of respondents, education level, type of dairy farm owners, training on dairy farm management, town or farm location, and herd size were significantly associated with biosecurity status. The majority of biosecurity measures were not applied, resulting in inadequate biosecurity implementation of the study dairy farms in central Ethiopia. Our findings indicate the presence of unacceptable biosecurity adoption by dairy farms. With this level of biosecurity, demographic factors of the dairy farmers showed associations with educational level, cooperatives found on dairy farming, lack of training on dairy farm management, and a shortage of land. As a result, intervention strategies, such as the provision of farmers' training on basic husbandry practices, and improved availability of necessary inputs are vitally important to curb the observed gaps.

## Data availability statement

The raw data supporting the conclusions of this article will be made available by the authors, without undue reservation.

## Author contributions

NM, KA, MTC, BM, and MD performed study conception, questionnaire development, and design of the study. NM and RK carried out data collection. NM, KA, BM, and HW performed data analysis and interpretation, and preparation of draft manuscript. All authors made critical revisions to the manuscript and approved the final version.
